# P-2158. Early Candida Isolation and Long-Term Survival After Lung Transplantation: Species-Specific Risks and Predictors

**DOI:** 10.1093/ofid/ofaf695.2321

**Published:** 2026-01-11

**Authors:** Scott A Cohen, Kathryn DeSear, Mattia Prosperi, Cynthia Gries

**Affiliations:** University of Florida, Gainesville, FL; UF health Shands Hospital, Gainesville, Florida; University of Florida, Gainesville, FL; University of Florida, Gainesville, FL

## Abstract

**Background:**

*Candida* species are common nosocomial or donor-derived fungal pathogens in lung transplant recipients, but their impact on outcomes, including species-specific variation, remain unclear. Given evolving fungal epidemiology, we evaluated the association between early *Candida* isolation with post-transplant survival, and clinical predictors of infection.

**Methods:**

We conducted a retrospective cohort study of incident lung transplant recipients at a tertiary-care center between 2010 to 2024. Eligible recipients were identified by procedural codes and associated hospitalization. Post-transplant *Candida* isolation was defined as any culture-positive isolation using microbiology and susceptibility testing reports. Follow-up began on the date of lung transplant, and the primary outcome was 10-year overall survival. Cox proportional hazards models, adjusted for demographics and transplant-specific factors, were applied to assess differences in pathogen isolation and survival. Logistic regression identified pre-transplant predictors of Candida isolation.

**Results:**

Among 463 lung transplant recipients, adjusted mortality rates were 7.3% (n=34) at 1 year and 58.0% (n=160) at 10 years post-transplant, with a median follow-up of 5.3 years. *Candida* was documented in 45 (9.7%) and 66 (14.3%) recipients within 30 days and 1 year after transplant, respectively. Most common *Candida* species were *C. glabrata* (n=31, 6.7%), *C. parapsilosis* (n=15, 3.2%), and *C. albicans/dubliniensis* (n=14, 3.0%). No *C. auris* was identified. Those with *Candida* isolation within 1 year after transplant had a significantly poorer survival (adjusted hazard ratio: 1.6, 95%CI: 1.0–2.5, p = 0.041). *C. glabrata* isolated within 30 days was associated with 2.1-fold increased risk of mortality (95%CI:1.1-4.0, p=0.026). Pre-transplant predictors of *C. glabrata* isolation within 30 days include history of myocardial infarction (adjusted odds ratio [aOR]: 7.7, p=0.012) and moderate/severe liver disease (aOR: 5.9, p=0.016).

**Conclusion:**

*Candida* isolation, particularly early *C. glabrata*, may be associated with reduced post-transplant survival. Specific baseline comorbidities predicted risk of infection. Future research will evaluate the impact of antifungal prophylaxis strategies on clinical outcomes.

**Disclosures:**

Kathryn DeSear, PharmD, Abbvie: Advisor/Consultant|Biomerieux: Advisor/Consultant|Cormedix: Speaking|GSK: Advisor/Consultant|Shionogi: Speaking

